# Assessment of computational methods for predicting the effects of missense mutations in human cancers

**DOI:** 10.1186/1471-2164-14-S3-S7

**Published:** 2013-05-28

**Authors:** Florian Gnad, Albion Baucom, Kiran Mukhyala, Gerard Manning, Zemin Zhang

**Affiliations:** 1 Department of Bioinformatics and Computational Biology, Genentech Inc., South San Francisco, CA 94080, USA

## Abstract

**Background:**

Recent advances in sequencing technologies have greatly increased the identification of mutations in cancer genomes. However, it remains a significant challenge to identify cancer-driving mutations, since most observed missense changes are neutral passenger mutations. Various computational methods have been developed to predict the effects of amino acid substitutions on protein function and classify mutations as deleterious or benign. These include approaches that rely on evolutionary conservation, structural constraints, or physicochemical attributes of amino acid substitutions. Here we review existing methods and further examine eight tools: SIFT, PolyPhen2, Condel, CHASM, mCluster, logRE, SNAP, and MutationAssessor, with respect to their coverage, accuracy, availability and dependence on other tools.

**Results:**

Single nucleotide polymorphisms with high minor allele frequencies were used as a negative (neutral) set for testing, and recurrent mutations from the COSMIC database as well as novel recurrent somatic mutations identified in very recent cancer studies were used as positive (non-neutral) sets. Conservation-based methods generally had moderately high accuracy in distinguishing neutral from deleterious mutations, whereas the performance of machine learning based predictors with comprehensive feature spaces varied between assessments using different positive sets. MutationAssessor consistently provided the highest accuracies. For certain combinations metapredictors slightly improved the performance of included individual methods, but did not outperform MutationAssessor as stand-alone tool.

**Conclusions:**

Our independent assessment of existing tools reveals various performance disparities. Cancer-trained methods did not improve upon more general predictors. No method or combination of methods exceeds 81% accuracy, indicating there is still significant room for improvement for driver mutation prediction, and perhaps more sophisticated feature integration is needed to develop a more robust tool.

## Background

Cancer arises as a result of genetic and epigenetic alterations in the genome. While most DNA mutations are considered neutral passenger mutations, driver mutations can increase the fitness of a cancer cell allowing its clonal expansion. Identifying driver mutations is crucial to elucidating tumorigenesis and revealing novel therapeutic targets. Recent developments in next-generation sequencing technologies enable extensive identification of DNA mutations in cancer as well as normal genomes. Large-scale efforts such as the Cancer Genome Atlas [1] have uncovered tens of thousands of sequence variants. While the avalanche of sequence data has revealed the spectrum of genetic variations in cancer, the results are difficult to interpret, as the vast majority of mutations do not drive tumorigenesis. Non-synonymous changes (those that change protein sequences) are the most investigated group of genetic perturbations. These mutations vary greatly in their functional impact, depending on their position and function in the protein and nature of the replacement amino acid. Several computational methods have been developed to predict the effect of any missense mutation on protein function, using evolutionary sequence comparison, structural constraints, and physicochemical attributes of amino acids. More recently, machine learning methods aim to specifically predict cancer-driving deleterious mutations, based on a wider set of attributes and training with sets of likely cancer mutations. These mutations form a subset of deleterious mutations in that they are positively selected during tumor evolution, but are negatively selected during organismal evolution. Metapredictors that combine several methods have also been developed [2].

In this study, we introduce and compare the results of several general and cancer-focused methods, using both known and novel testing sets. We discuss their individual strengths and highlight associated challenges as well as future prospects. We also examine the availability, coverage and inter-dependence of various tools.

## Materials and methods

### Datasets

We created a positive (non-neutral) test set of likely cancer driver mutations from the COSMIC database (v58) [3]. From a total of 40,707 missense mutations, we picked 2,682 mutations (corresponding to 482 genes) found in at least two tumor samples as likely driver mutations (Additional file 1). Since COSMIC has been used to develop some of the methods reviewed, we also created a novel test set, from recurrent somatic mutations in colorectal carcinoma identified in a very recent study of the Cancer Genome Atlas Network [4]. 455 somatic missense mutations were found in at least two tumor samples but not seen in COSMIC or dbSNP [5]. A second novel set of 147 recurrent unique mutations found in breast [6,7] or colon cancer [8] was similarly created.

Our negative (neutral) set of likely non-deleterious variants was built from germline SNPs found in dbSNP (Build Id 135). To avoid rare deleterious mutations and errors, we selected only SNPs with a minor allele frequency of at least 0.25, resulting in a set of 7,170 variants.

### Running predictors

We obtained SIFT 4.0.4 from http://sift.jcvi.org and followed the default instructions to install and run. A Java based pipeline was implemented to manage input and output data. We obtained PolyPhen-2 from http://genetics.bwh.harvard.edu/pph2 and followed the standard instructions for installation. Condel scores for the combination of SIFT and PolyPhen-2 were calculated with a Perl program provided by Ensembl. We retrieved functional impact scores from MutationAssessor, using http://mutationassessor.org. LogRE scores were derived with a Java class to align wild-type and mutant protein sequences against Pfam protein domain models (version 25.0) [9] using HMMER 3.0 [10]. The differences (wild-type versus mutant) of resulting E-values were used to calculate LogRE scores. SNAP was installed and applied in coordination with its developers from the Technische Universitaet Muenchen. mCluster scores were calculated as described [11]. CHASM scores were derived with CRAVAT (http://www.cravat.us).

### ROC curves and specificity/sensitivity estimation

Receiver operating characteristic (ROC) curves are composed of points that reflect the trade-off between true positive rate (sensitivity) and false positive rate (1 - specificity) at varying threshold values. For each predictive method, the score range was divided into 1000 bins, for which the proportions of variants from the positive and the negative set above and below the given threshold were calculated. Variants that were not covered (scored) by a method were excluded from the evaluation of that particular method. To assure the same number of mutations in the positive and negative sets, for each tool assessment the size of the neutral set was adjusted to the resulting depth of the covered non-neutral set with a preference for variants with high minor allele frequencies.

To calculate specificity and sensitivity values for each tool, we used score cutoffs that yielded the highest accuracy as measured by the proportion of correctly classified variations to the total number of variants in the test set.

### Metaprediction

Following the methodology of the Condel score [2], we used the weighted average of normalized scores to combine multiple predictions into a unified classification. Basically, normalized scores of each included tool and associated weights are used to calculate unified consensus scores. While the normalization of scores is straightforward, the calculation of weights requires reference cumulative distributions of true positives and true negatives. For mutations that are classified as deleterious by an individual tool, the weight of the normalized score reflects the probability that mutations with higher scores are not false positives based on the reference set. This probability is used as weight and increases with the score. On the other hand, for mutations that are predicted as benign, the weight reflects the probability that the mutation is not a false negative. Therefore low probabilities imply low contributions to the consensus score. The calculation of weights is illustrated in Additional file 2. We used the COSMIC set and the dbSNP set as reference sets to create the underlying cumulative distributions for weight estimation. Raw scores of individual methods were normalized to values between 0 (neutral) to 1 (non-neutral). The weighted average score (WAS) is defined as:

$$WAS=\frac{\sum_{i}S_{i}*W_{i}}{\sum_{i}W_{i}},$$

$$\begin{array}{cc} where & W_{i}=1-Pt_{i}\;\left(if\;variant\;is\;classified\;as\;deleterious\;in\;the\;ith\;tool\right)\\ & W_{i}=1-Pd_{i}\;\left(if\;variant\;is\;classified\;as\;tolerated\;in\;the\;ith\;tool\right) \end{array}$$

S_i _is the normalized score as calculated by the i-th tool, while W_i _is the weight for the given classification. Weights were calculated on the basis of the proportions (probabilities) of tolerated (Pt_i_) or deleterious (Pd_i_) variants with a normalized score higher than S_i _as observed for COSMIC mutations as positive set and dbSNP mutations as negative set.

## Results and discussion

### Overview of general tools for predicting the functional impact of amino acid changes

**Figure 1:**
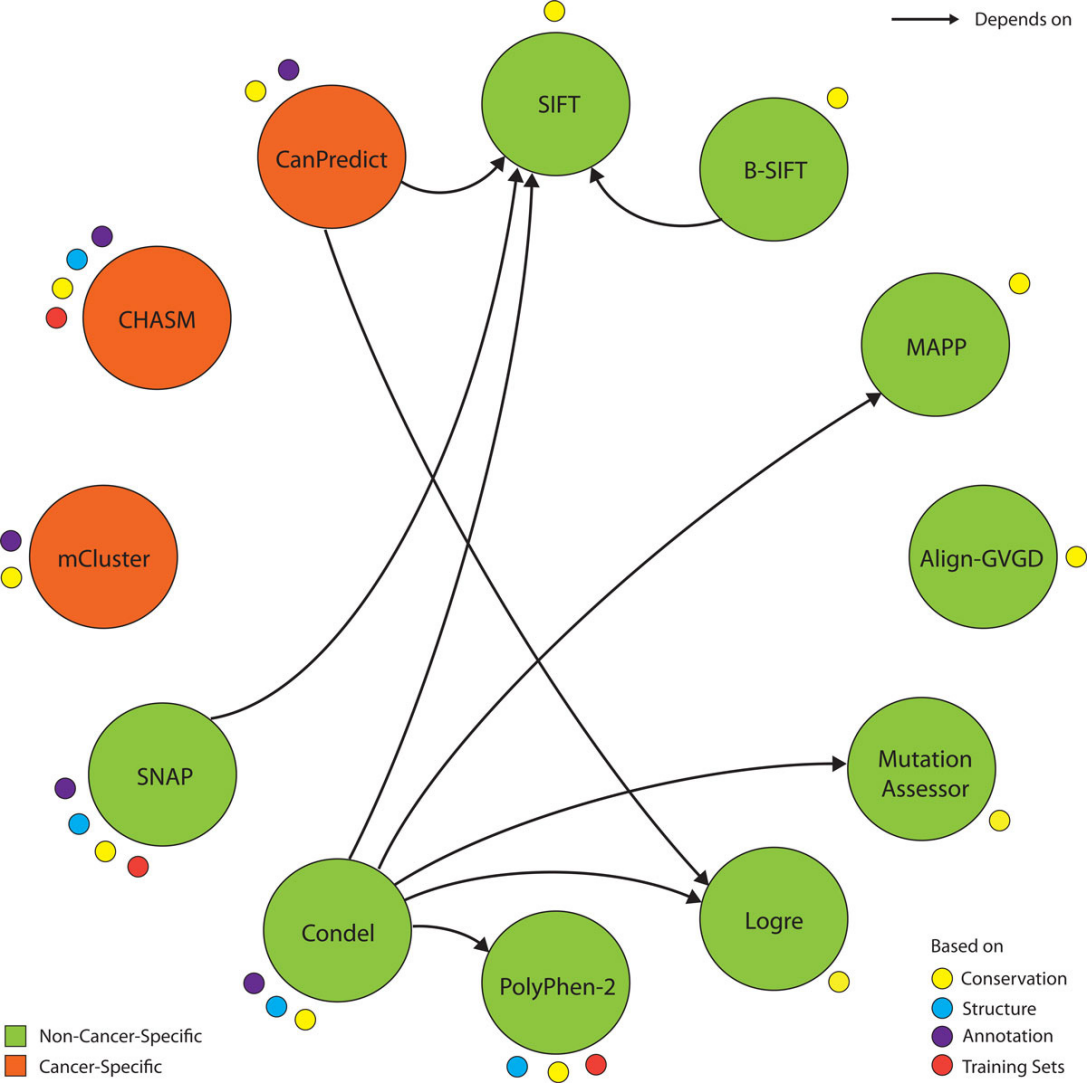
**Overview of representative predictors**. Predictors are annotated with the basis of their predictions, their cancer-specificity and reliance on each other. The pioneering SIFT method uses conservation information to predict the functional impact of amino acid changes. Several other approaches integrate SIFT results (arrows pointing to SIFT). The power of evolutionary information as an input feature is reflected by the number of classifiers that use conservation for prediction. For example, MAPP, SIFT, Align-GVGD, MutationAssessor and LogRE are predominantly based on conservation. PolyPhen-2 additionally integrates structure to classify mutations as deleterious or benign. Consensus classifiers such as Condel combine multiple predictive tools. The neural network-based SNAP represents one of several recently developed methods that rely on training sets and a large set of discriminatory features. Cancer-specific tools such as mCluster are specifically designed to identify driver mutations and also depend on mutation training sets. The machine learning based method CHASM spans an extensive feature space and is trained on canonical cancer driver mutations. In addition to evolutionary information, CanPredict takes into account gene ontology annotation for classifying oncogenes.

Most algorithms for predicting the functional impact of non-synonymous mutations are based on the observation that evolutionary and structural constraints are non-randomly distributed on proteins. This is consistent with the stronger conservation of functionally important residues and the higher probability of damaging mutations to occur in the protein interior [12]. Here we review some representative approaches (Figure 1) to provide some background for our assessment:

SIFT (Sorting Intolerant From Tolerant) [13,14] is a widely used pioneering method for identifying deleterious mutations using only evolutionary information. Installation and usage are straightforward, and the method depends only on PSI-BLAST [15]. SIFT identifies conserved protein residues based on multiple sequence alignment of homologous proteins, and calculates the probability for each of the 19 amino acid changes to be tolerated relative to the most frequent residue. Mutations of highly conserved protein positions tend to be predicted as deleterious, whereas changes in lower conserved protein regions are more likely to be neutral. Bi-directional SIFT (B-SIFT) [16] is a modification of SIFT that attempts to classify both gain- and loss-of-function mutations. By calculating SIFT scores for both the mutant and wild-type alleles, it identifies potential gain-of-function mutations where the mutant residue is more similar to those found in homologous proteins. As B-SIFT is exclusively based on SIFT, its implementation is also straightforward.

MutationAssessor [17] has a more elaborate conservation-based approach. It distinguishes between conservation patterns within aligned families (conservation score) and sub-families (specificity score) of homologs and so attempts to account for functional shifts between subfamilies of proteins. Specificity residues are defined by the clustering-based identification of homologous sequence subfamilies to determine functional specificity on the background of overall conservation. Interestingly, specificity residues were found to be predominantly located in binding interfaces on the protein surface implicating them in protein interaction [18].

In addition to conservation the feature space can be further increased by the inclusion of physiochemical characteristics. MAPP (Multivariate Analysis of Protein Polymorphism) [19,20] and Align-GVGD [21], for example, combine both evolutionary conservation and physiochemical information. While most sequence-based tools are capable of predicting the functional consequence of any mutation in a protein with homologs in other species, some are restricted to the classification of a subset of amino acid alterations. For example, LogRE (Log R Pfam E-value) [22] predicts only on Pfam domains, by comparing the Pfam score of the wild type and mutant alleles.

Structure-based methods model the structure of a protein using a protein structure database, and then examine structural features such as solvent accessibility or crystallographic B-factor surrounding the substituted amino acid. Predictors based exclusively on structural information have been clearly outcompeted. Their coverage is relatively low due to the lack of available protein structures, and the isolated context of a crystal structure might not reflect the functional importance of certain residues in an interactive environment. For example, a multitude of solvent accessible residues such as posttranslational modification sites are fundamental for protein function, which is reflected in their conservation [23,24], but not in their structural context. Combining sequence and structure information can increase prediction accuracy to a certain degree [25]. PolyPhen-2 [26] is the most prominent tool based on both sequence and structural information. It uses eight sequence-based and three structure-based features as input to a naive Bayes classification. Due to the diverse feature space, PolyPhen-2 is dependent on a variety of tools. For single amino acid substitutions it is therefore more straightforward to use the associated website (http://genetics.bwh.harvard.edu/pph2/).

To our knowledge the neural network-based tool SNAP (screening for non-acceptable polymorphisms) [27,28] spans the most comprehensive feature space. SNAP incorporates evolutionary constraints, structural features and protein annotation information. The most important single feature for SNAP prediction is conservation in a family of related proteins as reflected by PSIC scores [29]. As a result of the extensive feature space, SNAP depends on several other tools, which makes its installation complex. For a limited set of mutations it is possible to use SNAP's website.

These methods can give widely differing scores on the same variant, and have individual strengths and weaknesses. A combination of predictors may improve predictability. Condel (consensus deleteriousness score of missense mutations) [2] is a weighted average of the normalized scores from multiple methods. Implementing Condel is not complicated, but it involves the installation of various predictive methods and their supporting tools. Condel scores can be derived for a limited set of specified mutations via the corresponding web application, and the Ensembl database [30] provides position-specific Condel predictions that combine SIFT and Polyphen-2 for every possible amino acid substitution in all human proteins.

### Overview of cancer-specific predictors

Cancer driver mutations are a subset of deleterious mutations that decrease the organism's evolutionary fitness, while increasing cellular proliferation, survival or metastasis. Cancer-specific mutation predictors mainly use frequency-based or machine learning techniques trained on recurrent cancer mutations that are likely to be drivers. A variety of statistical methods has been developed to determine increased mutation frequency. mCluster [11] aggregates mutation data by mapping known disease related mutations to positions along conserved domains, and then mapping novel variants to those same conserved domains. Conserved mutation-enriched domain regions reflect hotspots for cancer driving functional changes. The mCluster score expresses the probability of observing a cluster of certain size given the number of positions in the domain and the mutation frequency. As a consequence of the underlying methodology, only mutations that occur in protein domains can be scored.

CHASM (cancer-specific high-throughput annotation of somatic mutations) [31] is a major machine learning approach that uses a random forest approach and is trained on cancer mutations from COSMIC and other cancer-related resources. CHASM uses an extensive set of 49 predictive features ranging from exon conservation to UniProt annotation [32] and frequency of missense change type in COSMIC. Notably the latter feature was ranked as second most predictive feature. CHASM is available via the web application CRAVAT (http://www.cravat.us).

Analogously to Condel, CanPredict [33] uses a random forest classifier to combine results from different methods. It uses SIFT and LogRE to determine the functional impact of changes, and Gene Ontology Similarity Score (GOSS) [34] to estimate the resemblance between the given mutated gene and known cancer-causing genes.

### Missense mutations from COSMIC and dbSNP used for testing

**Figure 2:**
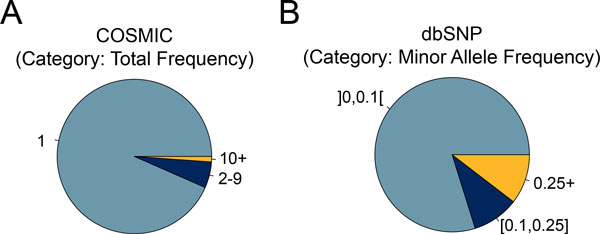
**Distribution of missense mutations in COSMIC and dbSNP**. (A) Most somatic non-synonymous mutations in COSMIC were identified in only one tumor sample. 7% of missense mutations were identified in two or more cancer samples. (B) In the dbSNP database global minor allele frequencies are provided for single nucleotide polymorphisms that were identified in the 1000 genomes project. 10% of the missense mutations have a minor allele frequency of 0.25 or higher, which increases their likelihood to be neutral.

To compare these methods, we created a positive test set of likely cancer driver mutations and a negative test set of likely benign variations (Materials and Methods). Few driver mutations have been well validated, so we used data from the COSMIC database of tumor-specific mutations. Most of these are random passenger mutations, but a substantial minority of positions are recurrently mutated: 2,682 of 40,707 positions are mutated 2 or more times (Figure 2) and are likely to be enriched for driver mutations. Common germline polymorphisms are likely to be largely neutral, so our negative test set consists of 7,170 variants in dbSNP with a reported minor allele frequency of at least 0.25.

**Figure 3:**
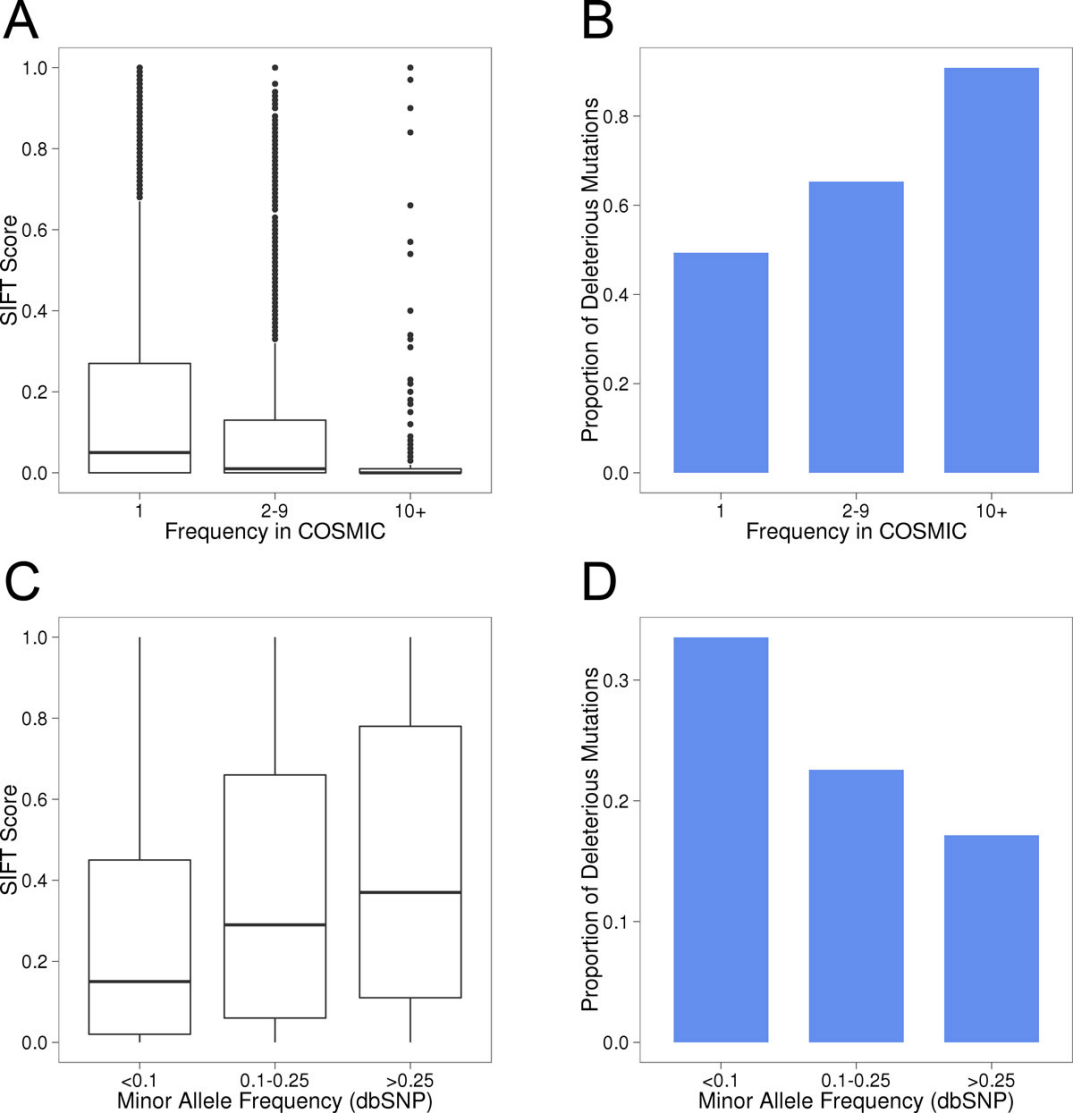
**SIFT predicts high frequency cancer mutations and low frequency SNPs to be more deleterious**. (A, B) The frequency of mutations in COSMIC correlates with the likelihood to be deleterious according to SIFT score (mutations that are predicted to be deleterious have low SIFT scores). (C, D) The minor allele frequency of dbSNP polymorphisms correlates with the likelihood to be benign according to SIFT score.

The criteria for selecting these datasets are supported by an initial scoring of all variants using SIFT. 49.4% of singleton COSMIC mutations score as deleterious (score<0.05), while 90.9% of mutations found in more than 10 samples score as deleterious (Figures 3a,b). In contrast, dbSNP variants with higher minor allele frequencies are predicted to be substantially more benign (Figures 3c,d). The same pattern was observed with PolyPhen-2 (Additional file 3).

Notably, these datasets do not represent a true gold standard in which all variants are either functionally deleterious or neutral, and there is in any case no uniform definition of functionality. However, they provide a sufficient enrichment in both classes of variants to be effective for comparison of methods. In general it is not straightforward to generate an optimal set for benchmark analysis. In contrast to the assessment of protein structure predictors, where the experimental structure gives a clear answer, the biology of underlying sets of missense mutations is far more complicated. We performed a relatively intuitive approach by taking recurrent somatic mutations as positive set. The overrepresentation of mutations of some canonical cancer genes in the COSMIC set supports our selection. For example, TP53, PTEN and EGFR each have more than 100 mutations reported in COSMIC.

### Coverage

**Figure 4:**
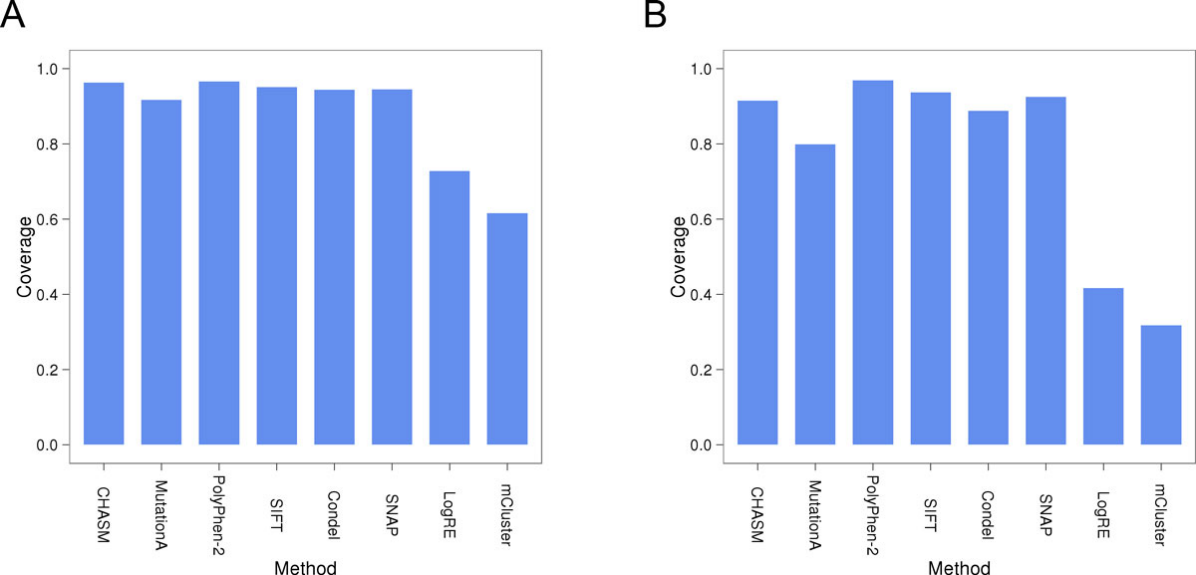
**Coverage of prediction**. CHASM, MutationA(ssessor), PolyPhen-2, SIFT, Condel, SNAP, and CHASM scored most missense mutations. LogRE and mCluster predictions are restricted to alterations that occur in domain regions and so scored less than 80% of likely cancer drivers (A). Coverage of likely-neutral mutations (B) was broadly similar, but with even lower coverage for LogRE and mCluster due to the lower prevalence of neutral mutations in domains.

We ran all predictors on both test sets (Materials and Methods). CHASM, MutationAssessor, PolyPhen-2, SIFT, Condel and SNAP were able to score most variants (Figure 4), each classifying at least 94% of COSMIC mutations. However, the reliability of predictions varies depending on the features scored. For example, with SIFT, low sequence diversity in the aligned homologs decreases classification confidence. Rare cases, where mutations could not be classified at all, can be explained by the absence of homologous proteins for evolutionary comparison. In contrast, LogRE and mCluster scored only 75% and 63% of cancer mutations respectively, since they can only predict within domain regions. They scored even fewer neutral variants (LogRE: 43%, mCluster: 33%), due to the relative scarcity of neutral mutations in domains. This limitation to classify only a specific group of variants can also be observed in other applications that are not further reviewed here. For example, approaches that are exclusively based on protein structures provide fewer predictions than conservation-based methods given the shortage of available protein structures opposed to the plethora of available sequence information.

### Prediction accuracy based on curated datasets

**Figure 5:**
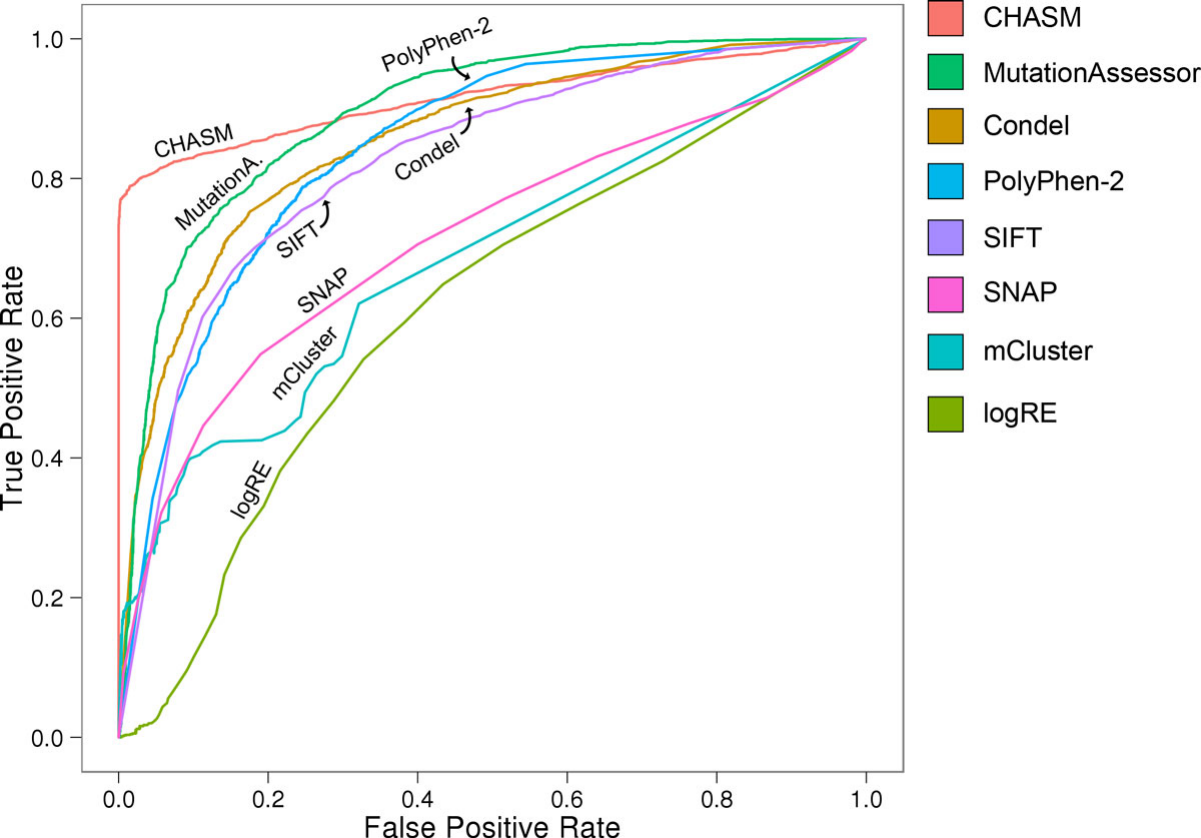
**Prediction accuracies compared between methods**. ROC curves for 8 predictors scored on COSMIC mutations and prevalent SNPs.

We compared prediction methods using a ROC analysis: using a range of score cutoffs to predict a mutation as deleterious, we plotted the fraction of likely drivers scored as deleterious ("True Positive Rate") against the fraction of likely benign variants scored as deleterious ("False Positive Rate") for a given score threshold (Figure 5, Table 1). Each method was scored with an equal number of neutral and cancer-associated variants.

**Table 1:** Prediction accuracies, sensitivities, specificities, AUC values and Matthew's correlation coefficients (MCC) compared between methods (based on COSMIC dataset)

Tool	Accuracy	Sensitivity	Specificity	AUC	MCC
CHASM	89%	79%	99%	0.92	0.79

MutationAssessor	81%	76%	86%	0.89	0.62

Condel	78%	75%	82%	0.85	0.58

PolyPhen-2	77%	79%	75%	0.82	0.54

SIFT	76%	70%	82%	0.80	0.52

SNAP	68%	55%	81%	0.67	0.37

mCluster	65%	40%	90%	0.64	0.35

logRE	61%	65%	57%	0.60	0.22

**Figure 6:**
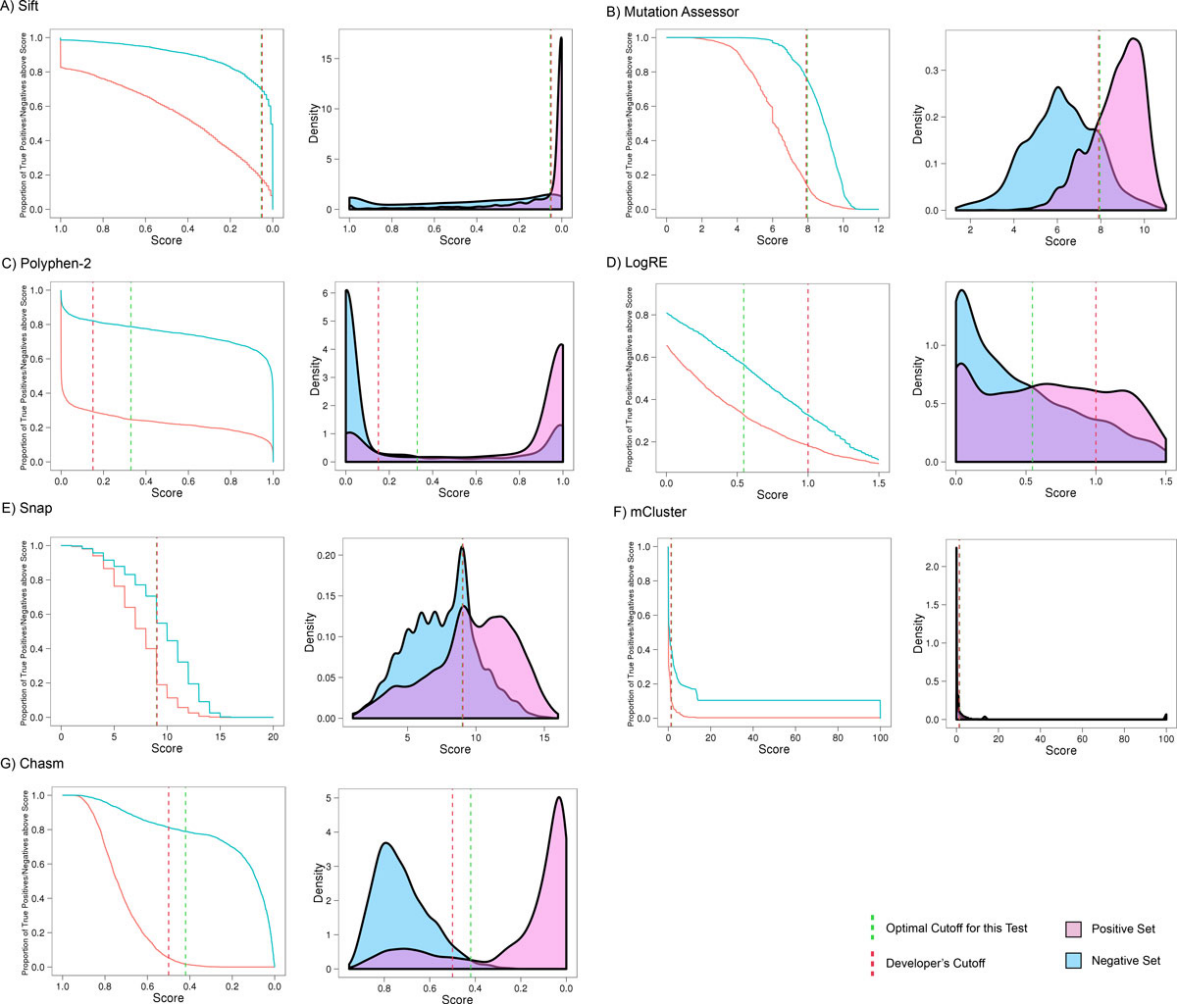
**Proportion of true positives and true negatives above certain score thresholds and corresponding score distributions**. Cumulative distributions of true positives and true negatives above certain score cutoffs form the basis for the derivation of weights for our metapredictors. In many cases calculated optimal cutoffs (marked in green) were similar to recommendations from the developers of the tools (marked in red). Both the cumulative distributions and the associated score distributions varied highly between the methods. We transformed raw scores of Snap and MutationAssessor, so that the minimum score is zero.

LogRE and mCluster were clearly outperformed by other methods. For LogRE, this agrees with a previous comparison [22]. mCluster, assumes that functionally important protein changes are enriched in conserved domain regions. The mCluster score of a given mutation increases with the frequency of all mutations from both the given dataset and curated disease-associated databases that occur in the same hotspot region. However, in our analysis the statistical power from the input set is depleted, as all mutations are counted as single events in our test set. Table 1 lists the sensitivity and specificity values calculated on the basis of score thresholds that yielded the highest accuracies as defined by the proportion of correctly classified variants in relation to the number of all variants in the test set (see also Additional file 4). In most cases the derived optimal cutoffs were similar to the thresholds recommended by the developers of the tools (Figure 6). In concordance with the resulting ROC curves, the accuracies of LogRE and mCluster were 61% and 65%, respectively.

In comparison, we found SIFT and PolyPhen-2 to have maximum accuracies of 76% and 77%, respectively. Saunders and Baker [25] showed that in general the additional inclusion of structural information (if available) contributes to a slight increase in performance. This might also play a role for the marginally increased performance of PolyPhen-2. The combination of Polyphen-2 and SIFT as reflected by the Condel score did not improve the accuracy significantly (78%).

**Figure 7:**
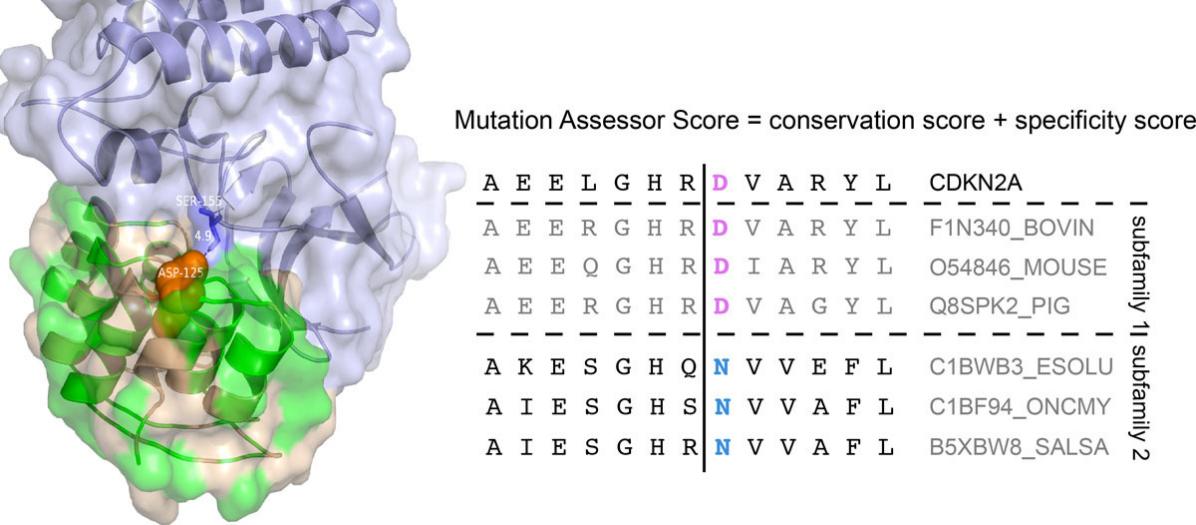
**Somatic mutation in CDKN2A predicted to be deleterious by MutationAssessor**. MutationAssessor predicted the somatic D125N mutation in the canonical tumor suppressor CDKN2A to be deleterious, due to its conservation in mammalian orthologs (Subfamily 1). Other tools used a wider array of homologs, including fish orthologs, where the residue is in fact N, and so classified the mutation to be benign. Using PyMOL (Version 1.2r3pre, Schrödinger, LLC.) the protein structure (PDB id: 1BI7) illustrates CDKN2A (wheat color) in complex with CDK6 (blue). The majority of residues in CDKN2A are known to be implicated in cancer based on UniProt (http://www.uniprot.org) annotation (green). D125 is shown in orange.

With an accuracy of 81% MutationAssessor yielded the second highest specificity across all methods at any sensitivity. As reviewed above, the use of evolutionary information in MutationAssessor differs from other sequence-based predictors. The methodology includes a refined class of conserved residues, termed specificity residues, to identify functional specificity on the background of overall conservation. Specificity residues are conserved within a subfamily but differ between subfamilies presumably encoding functional diversity. For example, a D125N mutation in CDKN2A (cyclin-dependent kinase inhibitor 2A) from liver cancer is scored as deleterious by MutationAssessor, because this residue is absolutely conserved as D in mammalian homologs (Figure 7, subfamily 1), but is scored as neutral by other methods that include more distant homologs, such as those of fishes, where the wild-type residue is N. The co-crystal of CDKN2A with cyclin-dependent kinase-6 (CDK6) shows that D125 is at the binding interface of the two proteins, close to Serine 155 (4.9A) of CDK6. Loss of this negative charge in the D125N mutant may substantially alter the binding affinity and so promote tumorigenesis.

Interestingly the accuracy of SNAP (68%) was lower than those of SIFT and PolyPhen-2, despite its more elaborate feature set. CHASM (89%) was the only tool that outperformed MutationAssessor in this assessment. CHASM predicted 99% of the negative set as non-drivers. However, recurrent COSMIC mutations were used to train the CHASM predictor, and several properties in CHASM's complex feature space are derived from COSMIC. For this reason, the CHASM performance in this test should be viewed with caution.

Excluding CHASM, the results of this assessment suggest that conservation based predictors, MutationAssessor in particular, achieve the highest accuracies in distinguishing neutral from deleterious mutations. However, none of these methods gives correct classifications of all mutations in the test sets. As an example for likely misclassification, MutationAssessor predicted the somatic G1007D mutation in phosphatidylinositol-4,5-biphosphate 3-kinase (PIK3CA), which was identified in haematopoietic, lymphoid and thyroid cancer, to be neutral, while all other methods defined the amino acid change to be deleterious. On the other hand, Bromberg and Rost showed that SNAP, which achieved relatively low sensitivity but high specificity in our assessment, outperformed competing approaches when using an independent dataset from four proteins (LacI repressor, bacteriophage T4 lysozyme, HIV-1 protease and human Melanocortin-4 receptor) [27]. Difference in performance might reflect testing dataset bias, or that cancer mutations are inherently different from those enzyme mutations commonly used in various training and testing programs.

### Prediction accuracy based on novel recurrent somatic mutations

**Figure 8:**
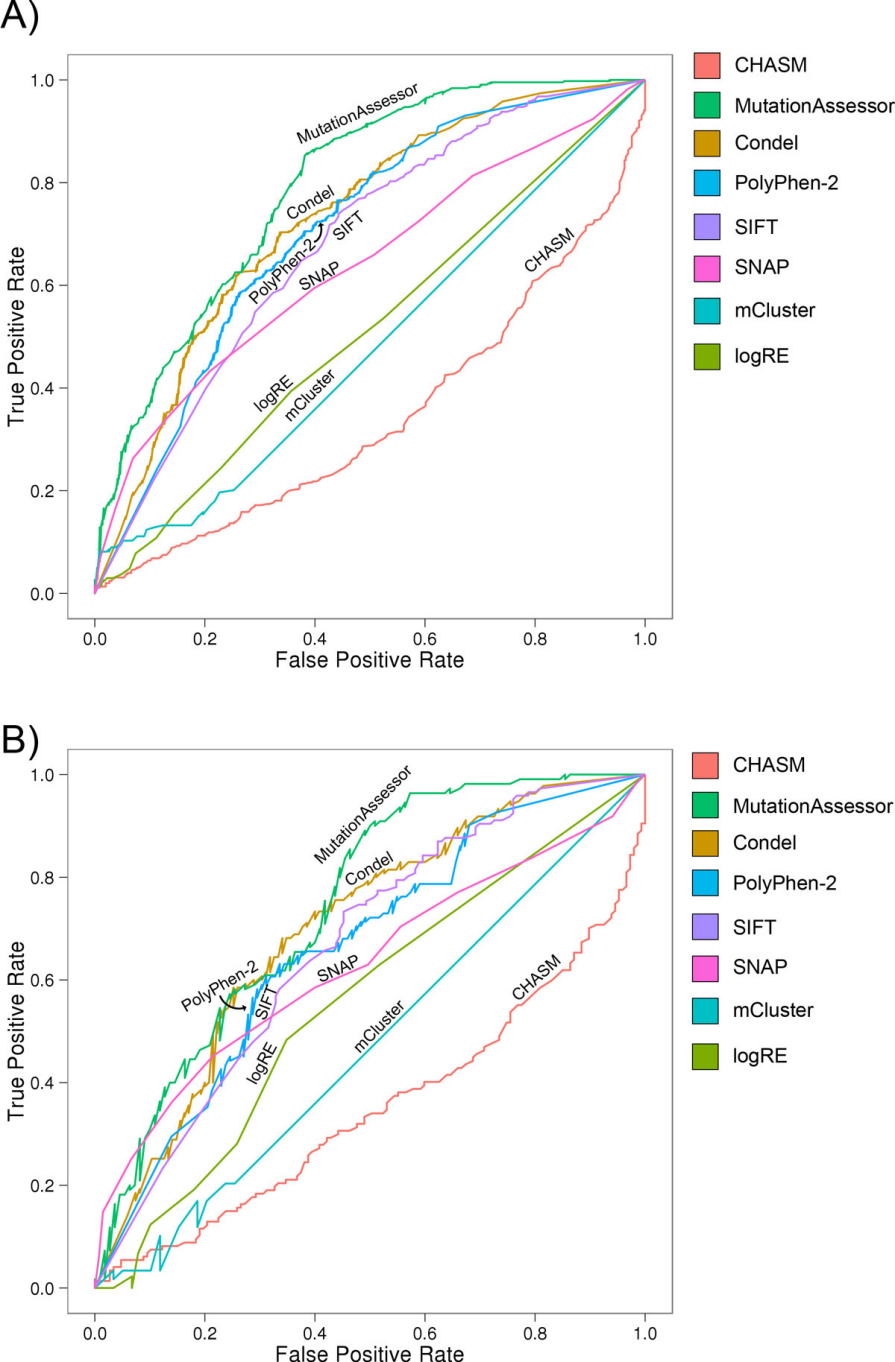
**Prediction accuracies based on novel recurrent somatic mutations**. (A) ROC curves for recurrent mutations found in the TCGA set. (B) ROC curves for recurrent mutations observed in the COBR set.

Since CHASM was explicitly trained on COSMIC mutations, and other methods may have been refined with it, we created new, independent positive test sets, of newly-identified recurrent mutations in colorectal tumors (2012a) ('TCGA' set) as well as recurrent somatic mutations in colon [8] or breast cancer [6,7] ('COBR' set) (Materials and Methods). We measured accuracy of each method on these new data and found similar results, with some notable differences (Figure 8):

Overall, we see a slight drop in prediction accuracy. This may be due to a drop in the severity of mutations in these new sets, since they exclude highly recurrent mutations seen in COSMIC. The most notable change is that CHASM accuracy dropped from 89% to 50%, as all mutations from the positive set were predicted to be neutral. The reason for this drop is not clear, but it has to be noted that mutations matching to COSMIC variants were ignored in this evaluation and these excluded mutations were the ones with the highest frequencies in the test sets. It should also be noted that the CHASM algorithm was developed to predict both tumor suppressor mutations as well as oncogene mutations. In our particular test, our choice of using recurrent mutation biased the data toward oncogenic driver mutations, which might contribute to the poor performance by CHASM. Furthermore, it is important to note that the relative frequency of missense changes in the COSMIC database is one of the 49 features used for CHASM prediction. Remarkably, this feature was shown to be the second most important feature for CHASM prediction. We purposely exclude any known COSMIC mutations in our independent test data, presumably causing the sharp performance drop by CHASM. It would be interesting to determine whether the CHASM performance might be more consistent across multiple test data sets if the COSMIC mutation frequency is excluded from the 49 feature collections.

The prediction accuracies of the other methods dropped to a lower degree, but their relative rankings were consistent with findings from the COSMIC set. The accuracy of PolyPhen-2 decreased from 77% for the COSMIC set to 66% and 65% for the TCGA set and the COBR set respectively, but achieved higher accuracies than SIFT or SNAP. For the TCGA set, Condel - as a combination of PolyPhen-2 and SIFT - marginally increased the accuracy from 66% and 65% respectively to 68%, and we found the same tendency for the COBR set (Figure 8, Tables 2 and 3). Notably, MutationAssessor performed best, with accuracies of 74% and 70% for the TCGA and COBR set, respectively.

**Table 2:** Prediction accuracies, sensitivities, specificities, AUC values and Matthew's correlation coefficients (MCC) compared between methods (based on TCGA dataset)

Tool	Accuracy	Sensitivity	Specificity	AUC	MCC
CHASM	50%	0%	100%	0.34	0.05

MutationAssessor	74%	86%	62%	0.79	0.49

Condel	68%	66%	66%	0.72	0.37

PolyPhen-2	66%	76%	56%	0.68	0.34

SIFT	65%	74%	56%	0.66	0.30

SNAP	62%	43%	79%	0.59	0.26

mCluster	54%	8%	99%	0.50	0.17

logRE	52%	39%	64%	0.50	0.07

**Table 3:** Prediction accuracies, sensitivities, specificities, AUC values and Matthew's correlation coefficients (MCC) compared between methods (based on COBR dataset)

Tool	Accuracy	Sensitivity	Specificity	AUC	MCC
CHASM	50%	0%	100%	0.36	0.08

MutationAssessor	70%	91%	50%	0.74	0.46

Condel	66%	66%	66%	0.68	0.33

PolyPhen-2	65%	63%	66%	0.63	0.30

SIFT	64%	73%	55%	0.63	0.29

SNAP	62%	45%	78%	0.59	0.26

mCluster	50%	0%	100%	0.46	0

logRE	54%	44%	64%	0.53	0.08

The observation that performances of individual methods can vary extremely between different test sets, is in concordance with findings from the Critical Assessment of Genome Interpretation (CAGI) project (http://genomeinterpretation.org) - an analogous approach to the critical assessment of techniques for protein structure prediction (CASP) [35].

### Combining individual predictors

To determine if multiple methods can be combined into a unified classification, we implemented metapredictors on the basis of weighted average scores [2] (Materials and Methods). We used cumulative distributions of true and false positives from the COSMIC set as reference to estimate weights (Figure 6). To validate the consensus classification on a dataset different from the reference set, we used the two sets of novel mutations. For both test sets, the performances of Condel (combining Polyphen-2 and SIFT) and our metapredictor that combined PolyPhen-2 and SIFT predictions were almost identical (Additional file 5), even though underlying distributions for weight estimation and cutoff optimization were different.

We examined several combinations of predictors and found that unifying predictions from Polyphen-2 and MutationAssessor, SIFT and MutationAssessor, or Polyphen-2 and SIFT achieved better predictions compared to other combinations. However, none of the combinations improved significantly on the best included predictor, and no combination improved on MutationAssessor alone. This is in contrast to a previous report in which combining prediction results from LogRE, MAPP, Mutation Asssessor, PolyPhen-2 and SIFT was shown to outperform each individual method [2]. The reason of this difference is not clear, but it is possible that only certain datasets are suitable for metaprediction approaches.

## Conclusions

Our independent assessment of commonly available tools reveals challenges and inconsistencies of existing tools. Although the cancer-specific predictor CHASM performed particularly well using COSMIC mutations, we observed a dramatic drop in performance when using novel recurrent mutations not present in the COSMIC database. Other cancer-specific methods did not perform better than general tools for predicting the functional impact of amino acid changes. It is debatable what causes such performance difference. One major challenge is the generation of underlying datasets for training and testing. Using recurrent somatic changes as positive set seems to be an intuitive and reasonable approach. However, there is no experimental evidence for the potential to be driver mutations in cancer. It is clear that machine learning-based approaches are essentially affected by this problem and need further improvement to become generally applicable. In contrast, sequence conservation-based approaches seem to be less affected by different testing datasets. In fact, MutationAssessor provides consistently reasonable prediction results in this study. However, it is premature to declare any single predictor as the sole winner since we have identified many instances where an otherwise good predictor would completely miss obvious driver mutations. It is not obvious that metapredictors based on multiple approaches would produce the "silver bullet" cancer driver mutation predictor, therefore novel and more robust methodology development is still needed.

One idea for potential improvement is to train specialized predictors on different classes of putative driver mutations. Functional driver mutations can impact both tumor suppressors and oncogenes, and the characteristics of these mutations are epected to be different. While tumor suppressors are likely impacted by inactiving mutations, oncogenes can be impacted by a more complex pattern. Mutations that activate oncogenes may exert their effect by different mechanisms, such as utilizing residues that are evolutionarily more fit, inactiving a regulatory region to make a kinase constitutively active, or simulating the activated state of a protein. It is perhaps more practical to develop multiple specific algorithms for different classes of mutations, instead of develop a "one-size-fit-all" approach. With more validated, novel driver mutation data available, such robust and specialized prediction tools should be within reach.

## Competing interests

The authors declare that they have no competing interests.

## Authors' contributions

FG carried out the data analysis, programming and drafting the manuscript. AB helped to install and run predictors. GM helped to draft the manuscript. KM carried out the structural analysis of specific mutations. ZZ initiated and supervised the project, and helped to draft the manuscript.

## Supplementary Material

Additional file 1 **Datasets used for assessments**. Click here for file

Additional file 2 **Calculating the weights for metaprediction**. Following the methodology of the Condel score [2], we used the weighted average of the normalized scores to combine the results of multiple predictors into a unified consensus score. The weighted average score is calculated on the basis of normalized prediction scores and weights. Weights are estimated from cumulative distributions of true positives and true negatives above given scores. Click here for file

Additional file 3 **Distribution and proportion of missense mutations predicted to be deleterious by PolyPhen-2**. The frequency of somatic mutations in the COSMIC database correlates with the likelihood to be damaging according to PolyPhen-2 predictions (A, B). The global minor allele frequency of single nucleotide polymorphisms in the dbSNP database correlates with the likelihood to be benign according to PolyPhen-2 classifications (C, D). Click here for file

Additional file 4 **Calculating the optimal cutoff yielding the highest accuracy for each method**. Accuracy is defined as the proportion of true positives and true negatives in relation to all positives and negatives. The accuracy increases with the true positive rate (sensitivity) until the proportion of false positives outweighs. The peak of each curve reflects the optimal accuracy. The corresponding score thresholds were used to calculate specificity and sensitivity values for each method. Click here for file

Additional file 5 **Comparison of Condel and our metapredictor**. Based on an ROC analysis using the TCGA set (A) and the COBR set (B) as test sets, the performances of our metapredictor and Condel are almost identical. Both approaches combine PolyPhen-2 and SIFT predictions, but use different underlying reference sets for weight estimation. Click here for file

## Declarations

The costs for this article were covered by Genentech Inc.

This article has been published as part of *BMC Genomics *Volume 14 Supplement 3, 2013: SNP-SIG 2012: Identification and annotation of SNPs in the context of structure, function, and disease. The full contents of the supplement are available online at http://www.biomedcentral.com/bmcgenomics/supplements/14/S3
