# Supplementary figures and images for: Assessment of computational methods for predicting the effects of missense mutations in human cancers
Source: BMC Genomics. 2013 May 28;14(Suppl 3):S7. doi: 10.1186/1471-2164-14-S3-S7 (PMC3665581; doi:10.1186/1471-2164-14-S3-S7)

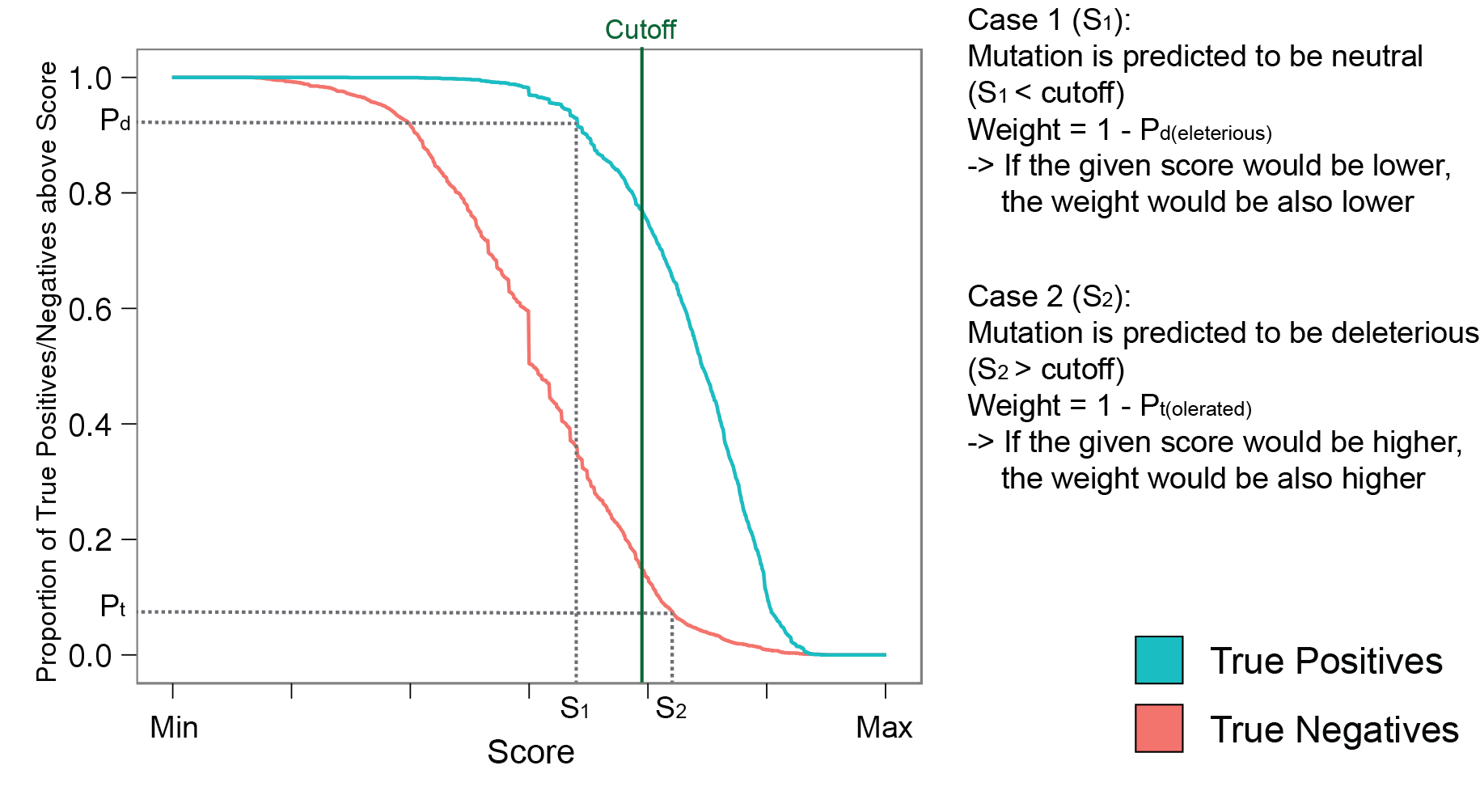

Supplement: Additional file 2 — Calculating the weights for metaprediction. Following the methodology of the Condel score [2], we used the weighted average of the normalized scores to combine the results of multiple predictors into a unified consensus score. The weighted average score is calculated on the basis of normalized prediction scores and weights. Weights are estimated from cumulative distributions of true positives and true negatives above given scores. [file 1471-2164-14-S3-S7-S2.png]

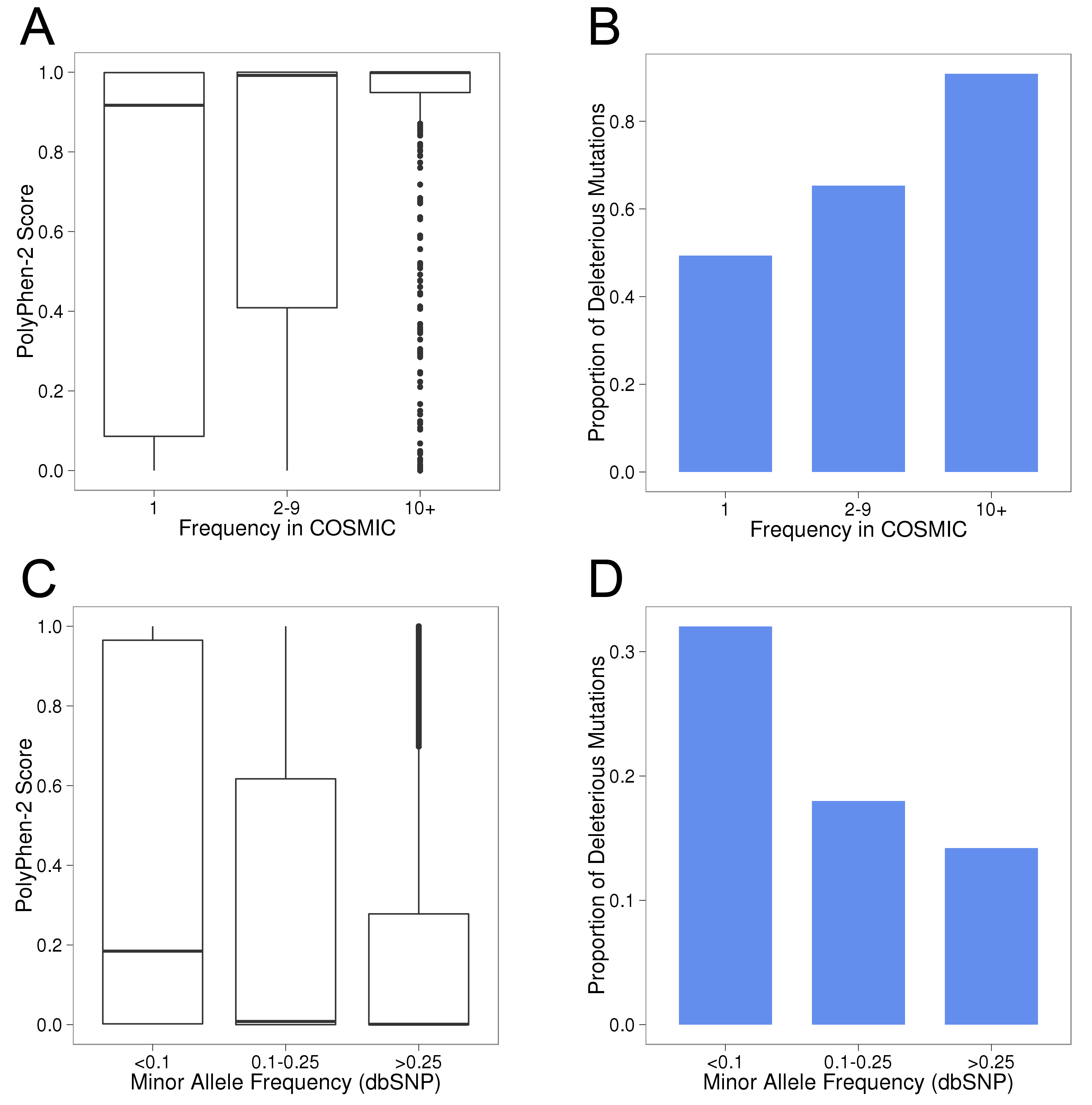

Supplement: Additional file 3 — Distribution and proportion of missense mutations predicted to be deleterious by PolyPhen-2. The frequency of somatic mutations in the COSMIC database correlates with the likelihood to be damaging according to PolyPhen-2 predictions (A, B). The global minor allele frequency of single nucleotide polymorphisms in the dbSNP database correlates with the likelihood to be benign according to PolyPhen-2 classifications (C, D). [file 1471-2164-14-S3-S7-S3.png]

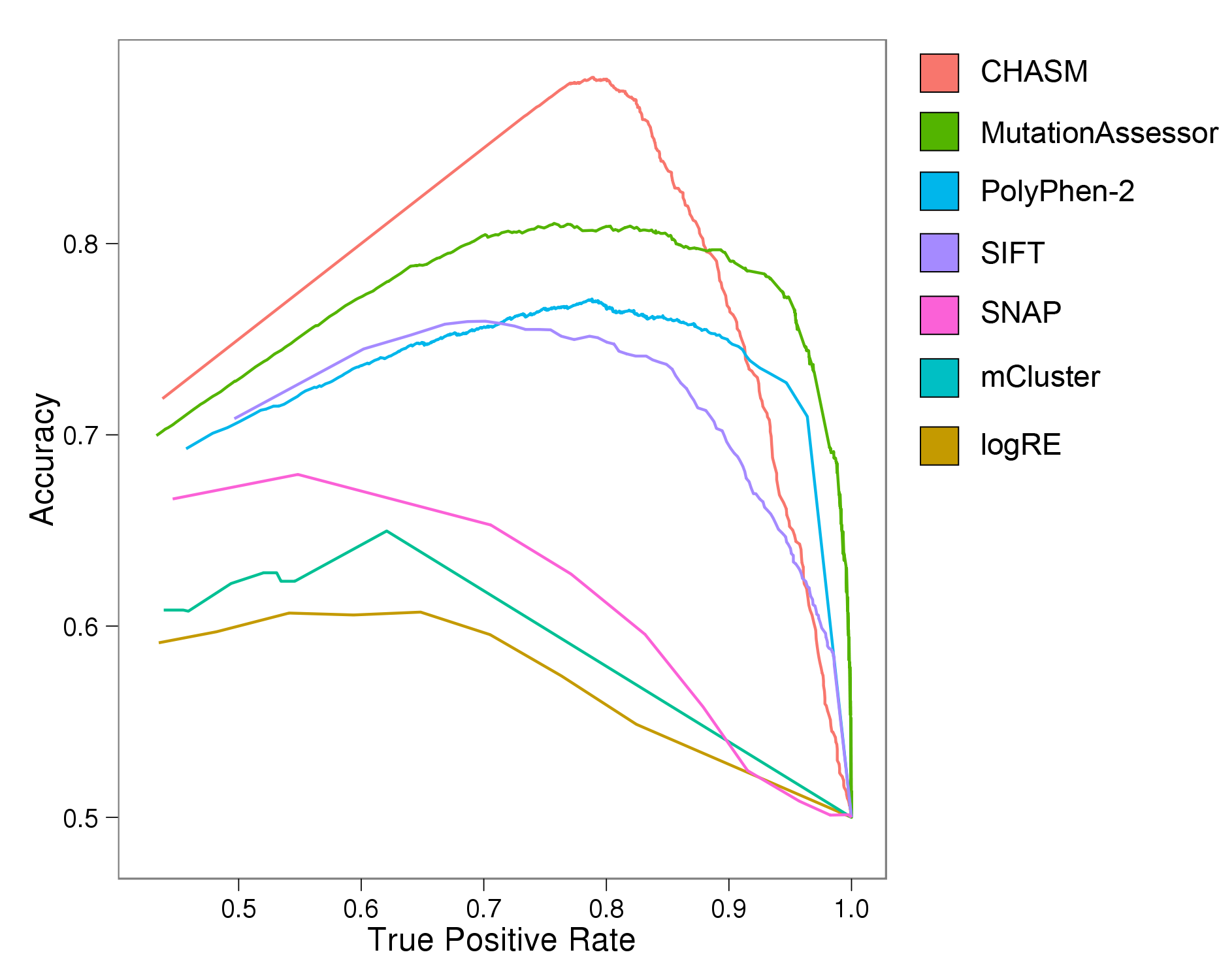

Supplement: Additional file 4 — Calculating the optimal cutoff yielding the highest accuracy for each method. Accuracy is defined as the proportion of true positives and true negatives in relation to all positives and negatives. The accuracy increases with the true positive rate (sensitivity) until the proportion of false positives outweighs. The peak of each curve reflects the optimal accuracy. The corresponding score thresholds were used to calculate specificity and sensitivity values for each method. [file 1471-2164-14-S3-S7-S4.png]

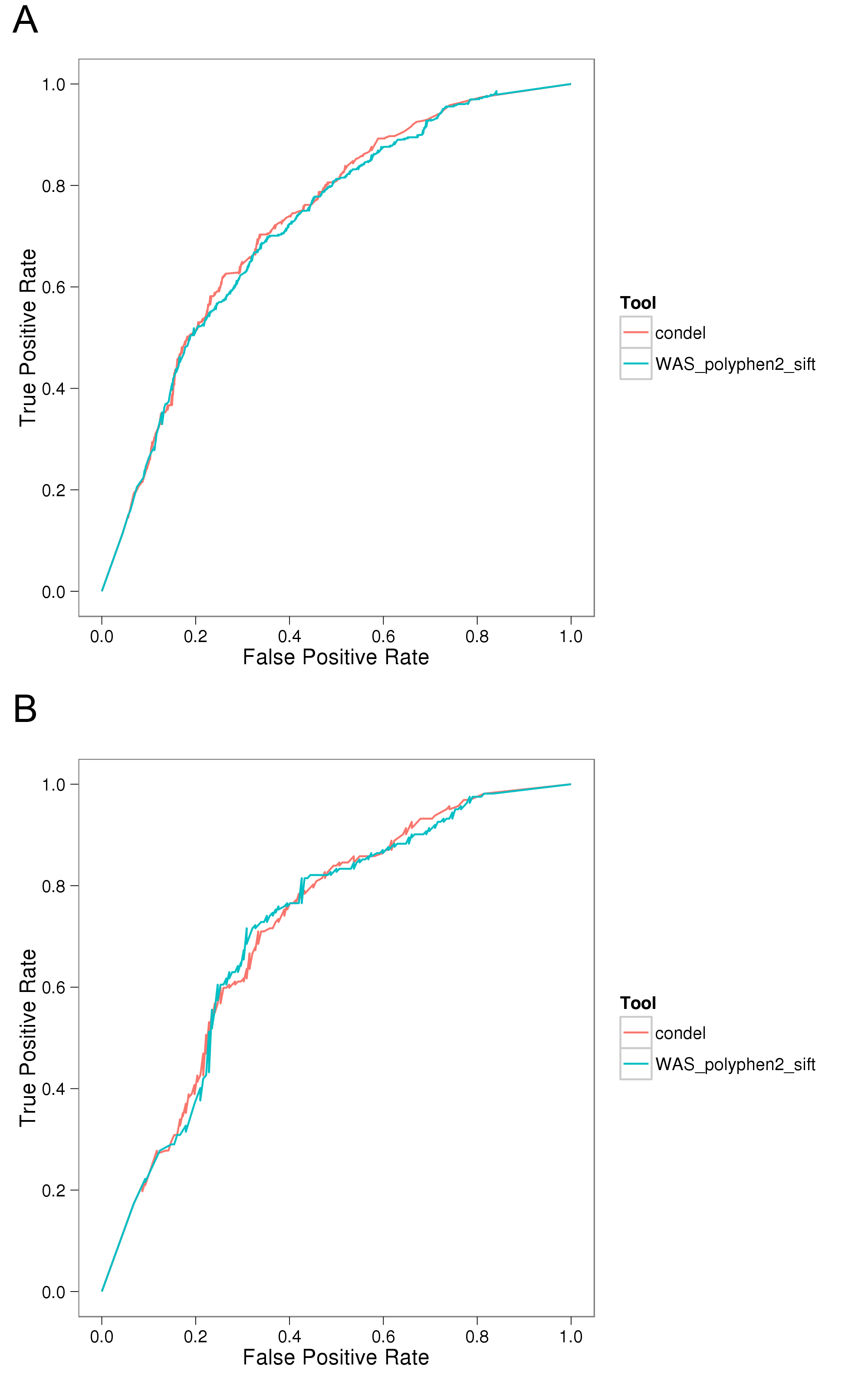

Supplement: Additional file 5 — Comparison of Condel and our metapredictor. Based on an ROC analysis using the TCGA set (A) and the COBR set (B) as test sets, the performances of our metapredictor and Condel are almost identical. Both approaches combine PolyPhen-2 and SIFT predictions, but use different underlying reference sets for weight estimation. [file 1471-2164-14-S3-S7-S5.png]
